# *catena*-Poly[[dimethyl­bis­(thio­cyanato-κ*N*)tin(IV)]-μ-(4,4′-bipyridine-κ^2^ *N*:*N*′)]

**DOI:** 10.1107/S1600536811005459

**Published:** 2011-02-19

**Authors:** Ezzatollah Najafi, Mostafa M. Amini, Seik Weng Ng

**Affiliations:** a Department of Chemistry, General Campus, Shahid Beheshti University, Tehran 1983963113, Iran; b Department of Chemistry, University of Malaya, 50603 Kuala Lumpur, Malaysia

## Abstract

The title dimethyl­tin diisothio­cyanate adduct of 4,4′-bipyridine, [Sn(CH_3_)_2_(NCS)_2_(C_10_H_8_N_2_)]_*n*_, adopts a chain motif in which the *N*-heterocycle functions as a bridge to adjacent all-*trans* octa­hedrally coordinated tin atoms. The Sn^IV^ atom lies on a special position of 2/*m* site symmetry, the methyl C atom on a special position of 2 site symmetry, and the thio­cyanate and 4,4′-bipyridine on a special position of *m* site symmetry.

## Related literature

For the 4,4′-bipyridine adducts of diorganotin dichlorides, see: Ma *et al.* (2004 ▶); Ng (1998 ▶). For the dimethyl­tin di(isothio­cyanate) adduct of 1,10-phenanthroline, see: Najafi *et al.* (2011 ▶).
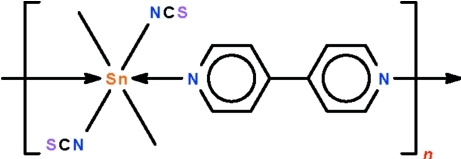


## Experimental

### 

#### Crystal data

[Sn(CH_3_)_2_(NCS)_2_(C_10_H_8_N_2_)]
*M* *_r_* = 421.10
Monoclinic, 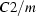

*a* = 10.8697 (8) Å
*b* = 7.7741 (6) Å
*c* = 11.3979 (8) Å
β = 115.817 (1)°
*V* = 867.0 (1) Å^3^
*Z* = 2
Mo *K*α radiation
μ = 1.71 mm^−1^
*T* = 295 K
0.30 × 0.20 × 0.10 mm

#### Data collection

Bruker SMART APEX diffractometer
Absorption correction: multi-scan (*SADABS*; Sheldrick, 1996 ▶) *T*_min_ = 0.628, *T*_max_ = 0.847
4033 measured reflections
1066 independent reflections
1064 reflections with *I* > 2σ(*I*)
*R*_int_ = 0.021

#### Refinement

*R*[*F* ^2^ > 2σ(*F* ^2^)] = 0.023
*wR*(*F* ^2^) = 0.061
*S* = 1.08
1066 reflections
64 parameters
H-atom parameters constrained
Δρ_max_ = 0.49 e Å^−3^
Δρ_min_ = −0.82 e Å^−3^

### 

Data collection: *APEX2* (Bruker, 2009 ▶); cell refinement: *SAINT* (Bruker, 2009 ▶); data reduction: *SAINT*; program(s) used to solve structure: *SHELXS97* (Sheldrick, 2008 ▶); program(s) used to refine structure: *SHELXL97* (Sheldrick, 2008 ▶); molecular graphics: *X-SEED* (Barbour, 2001 ▶); software used to prepare material for publication: *publCIF* (Westrip, 2010 ▶).

## Supplementary Material

Crystal structure: contains datablocks global, I. DOI: 10.1107/S1600536811005459/si2334sup1.cif

Structure factors: contains datablocks I. DOI: 10.1107/S1600536811005459/si2334Isup2.hkl

Additional supplementary materials:  crystallographic information; 3D view; checkCIF report

## Figures and Tables

**Fig. 1.**
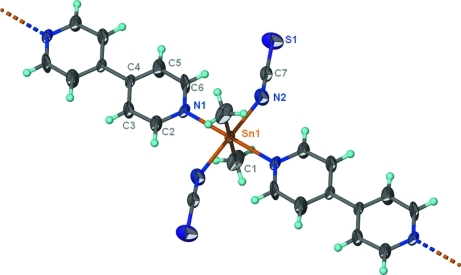
Thermal ellipsoid plot (Barbour, 2001) of a portion of the Sn(NCS)2(CH3)2(C10H8N2) chain at the 50% probability level. Hydrogen atoms are drawn as spheres of arbitrary radius.

## supplementary crystallographic information

### Comment

The 4,4'-bipyridine ligand forms a number of adducts with diorganotin dihalides; the adducts adopt linear chain structures as the ligand functions in a bridging mode. The organotin dihalides include dimethyltin dichloride (Ng, 1998), dibutyltin dichloride and dibenzyltin dichloride (Ma *et al.*, 2004); no pseudohalides have been reported. The dimethyltin diisothiocyanate adduct similarly adopts a chain motif (Scheme I, Fig. 1). Polymeric [Sn(NCS)_2_(CH_3_)_2_(C_10_H_8_N_2_)_2_]*_n_* has the *N*-heterocycle functioning as a bridging to adjacent all-*trans* octahedrally coordinated tin atoms. The tin atom lies on a special position of 2/*m* site symmetry, the methyl carbon on a special position of *2* site symmetry, and the isothiocyanate and 4,4'-bipyridine on a special position of *m* site symmetry. The geometry of the tin atom in the dimethyltin di(isothiocyanate) adduct with 1,10-phenanthroline is a *cis*-octahedron (Najafi *et al.*, 2011).

### Experimental

Dimethyltin diisothiocyanate (1 mmol, 0.26 g) and 4,4'-bipyridine (1 mmol, 0.16 g) were loaded into a convection tube. The tube was filled with acetonitrile and methanol (v:v / 9:1) and kept at 333 K. Colorless crystals were collected from the side arm after several days.

### Refinement

Hydrogen atoms were placed in calculated positions (C–H 0.93–0.96 Å) and were included in the refinement in the riding model approximation, with *U*_iso_(H) set to 1.2–1.5*U*_eq_(C).

### Crystal data

**Table:**

[Sn(CH_3_)_2_(NCS)_2_(C_10_H_8_N_2_)]	*F*(000) = 416
*M**_r_* = 421.10	*D*_x_ = 1.613 Mg m^−^^3^
Monoclinic, *C*2/*m*	Mo *K*α radiation, λ = 0.71073 Å
Hall symbol: -C 2y	Cell parameters from 3765 reflections
*a* = 10.8697 (8) Å	θ = 3.8–28.3°
*b* = 7.7741 (6) Å	µ = 1.71 mm^−^^1^
*c* = 11.3979 (8) Å	*T* = 295 K
β = 115.817 (1)°	Prism, colorless
*V* = 867.0 (1) Å^3^	0.30 × 0.20 × 0.10 mm
*Z* = 2	

### Data collection

**Table:**

Bruker SMART APEX diffractometer	1066 independent reflections
Radiation source: fine-focus sealed tube	1064 reflections with *I* > 2σ(*I*)
graphite	*R*_int_ = 0.021
ω scans	θ_max_ = 27.5°, θ_min_ = 3.4°
Absorption correction: multi-scan (*SADABS*; Sheldrick, 1996)	*h* = −14→13
*T*_min_ = 0.628, *T*_max_ = 0.847	*k* = −10→10
4033 measured reflections	*l* = −14→14

### Refinement

**Table:**

Refinement on *F*^2^	Primary atom site location: structure-invariant direct methods
Least-squares matrix: full	Secondary atom site location: difference Fourier map
*R*[*F*^2^ > 2σ(*F*^2^)] = 0.023	Hydrogen site location: inferred from neighbouring sites
*wR*(*F*^2^) = 0.061	H-atom parameters constrained
*S* = 1.08	*w* = 1/[σ^2^(*F*_o_^2^) + (0.048*P*)^2^ + 0.1158*P*] where *P* = (*F*_o_^2^ + 2*F*_c_^2^)/3
1066 reflections	(Δ/σ)_max_ = 0.001
64 parameters	Δρ_max_ = 0.49 e Å^−^^3^
0 restraints	Δρ_min_ = −0.82 e Å^−^^3^

### Fractional atomic coordinates and isotropic or equivalent isotropic displacement parameters (Å^2^)

**Table:**

	*x*	*y*	*z*	*U*_iso_*/*U*_eq_	Occ. (<1)
Sn1	0.5000	0.5000	0.5000	0.03309 (11)	
S1	0.6834 (2)	0.5000	0.18029 (16)	0.0967 (5)	
N1	0.3011 (3)	0.5000	0.2981 (2)	0.0404 (5)	
N2	0.6379 (4)	0.5000	0.3960 (4)	0.0763 (12)	
C1	0.5000	0.2290 (5)	0.5000	0.0713 (12)	
H1A	0.5254	0.1878	0.5869	0.107*	0.50
H1B	0.5644	0.1878	0.4697	0.107*	0.50
H1C	0.4103	0.1878	0.4434	0.107*	0.50
C2	0.1780 (4)	0.5000	0.2946 (3)	0.0713 (14)	
H2	0.1717	0.5000	0.3734	0.086*	
C3	0.0584 (4)	0.5000	0.1812 (3)	0.0721 (15)	
H3	−0.0254	0.5000	0.1849	0.087*	
C4	0.0628 (3)	0.5000	0.0623 (3)	0.0388 (6)	
C5	0.1893 (4)	0.5000	0.0663 (4)	0.102 (3)	
H5	0.1985	0.5000	−0.0112	0.122*	
C6	0.3047 (4)	0.5000	0.1840 (4)	0.099 (2)	
H6	0.3896	0.5000	0.1826	0.119*	
C7	0.6565 (3)	0.5000	0.3071 (4)	0.0501 (8)	

### Atomic displacement parameters (Å^2^)

**Table:**

	*U*^11^	*U*^22^	*U*^33^	*U*^12^	*U*^13^	*U*^23^
Sn1	0.02266 (15)	0.04968 (17)	0.02072 (15)	0.000	0.00366 (10)	0.000
S1	0.1031 (11)	0.1490 (14)	0.0594 (8)	0.000	0.0554 (8)	0.000
N1	0.0246 (11)	0.0643 (15)	0.0225 (11)	0.000	0.0011 (9)	0.000
N2	0.0413 (17)	0.146 (4)	0.0449 (19)	0.000	0.0216 (16)	0.000
C1	0.064 (3)	0.0532 (19)	0.068 (3)	0.000	0.002 (2)	0.000
C2	0.0298 (16)	0.154 (5)	0.0201 (15)	0.000	0.0018 (13)	0.000
C3	0.0252 (16)	0.157 (5)	0.0280 (17)	0.000	0.0057 (14)	0.000
C4	0.0231 (13)	0.0624 (16)	0.0215 (13)	0.000	0.0009 (12)	0.000
C5	0.0260 (17)	0.253 (8)	0.0205 (17)	0.000	0.0053 (14)	0.000
C6	0.0210 (16)	0.244 (8)	0.0231 (17)	0.000	0.0016 (14)	0.000
C7	0.0300 (15)	0.077 (2)	0.0387 (17)	0.000	0.0110 (13)	0.000

### Geometric parameters (Å, °)

**Table:**

Sn1—C1^i^	2.107 (4)	C1—H1B	0.9600
Sn1—C1	2.107 (4)	C1—H1C	0.9600
Sn1—N2^i^	2.280 (3)	C2—C3	1.378 (5)
Sn1—N2	2.280 (3)	C2—H2	0.9300
Sn1—N1^i^	2.374 (2)	C3—C4	1.376 (4)
Sn1—N1	2.374 (2)	C3—H3	0.9300
S1—C7	1.595 (4)	C4—C5	1.356 (5)
N1—C6	1.318 (5)	C4—C4^ii^	1.480 (5)
N1—C2	1.321 (5)	C5—C6	1.381 (5)
N2—C7	1.116 (5)	C5—H5	0.9300
C1—H1A	0.9600	C6—H6	0.9300
			
C1^i^—Sn1—C1	180.0	H1A—C1—H1B	109.5
C1^i^—Sn1—N2^i^	90.0	Sn1—C1—H1C	109.5
C1—Sn1—N2^i^	90.000 (1)	H1A—C1—H1C	109.5
C1^i^—Sn1—N2	90.000 (1)	H1B—C1—H1C	109.5
C1—Sn1—N2	90.0	N1—C2—C3	123.9 (3)
N2^i^—Sn1—N2	180.000 (1)	N1—C2—H2	118.1
C1^i^—Sn1—N1^i^	90.000 (1)	C3—C2—H2	118.1
C1—Sn1—N1^i^	90.000 (1)	C4—C3—C2	120.0 (3)
N2^i^—Sn1—N1^i^	91.34 (12)	C4—C3—H3	120.0
N2—Sn1—N1^i^	88.66 (12)	C2—C3—H3	120.0
C1^i^—Sn1—N1	90.0	C5—C4—C3	115.9 (3)
C1—Sn1—N1	90.000 (1)	C5—C4—C4^ii^	122.0 (3)
N2^i^—Sn1—N1	88.66 (12)	C3—C4—C4^ii^	122.1 (4)
N2—Sn1—N1	91.34 (12)	C4—C5—C6	120.7 (3)
N1^i^—Sn1—N1	180.0	C4—C5—H5	119.6
C6—N1—C2	115.8 (3)	C6—C5—H5	119.6
C6—N1—Sn1	123.4 (2)	N1—C6—C5	123.6 (3)
C2—N1—Sn1	120.8 (2)	N1—C6—H6	118.2
C7—N2—Sn1	153.1 (3)	C5—C6—H6	118.2
Sn1—C1—H1A	109.5	N2—C7—S1	179.8 (4)
Sn1—C1—H1B	109.5		
			
C1^i^—Sn1—N1—C6	90.0	N1—Sn1—N2—C7	0.000 (2)
C1—Sn1—N1—C6	−90.0	C6—N1—C2—C3	0.0
N2^i^—Sn1—N1—C6	180.0	Sn1—N1—C2—C3	180.0
N2—Sn1—N1—C6	0.0	N1—C2—C3—C4	0.0
C1^i^—Sn1—N1—C2	−90.0	C2—C3—C4—C5	0.0
C1—Sn1—N1—C2	90.0	C2—C3—C4—C4^ii^	180.0
N2^i^—Sn1—N1—C2	0.0	C3—C4—C5—C6	0.0
N2—Sn1—N1—C2	180.0	C4^ii^—C4—C5—C6	180.0
C1^i^—Sn1—N2—C7	−90.000 (1)	C2—N1—C6—C5	0.0
C1—Sn1—N2—C7	90.000 (1)	Sn1—N1—C6—C5	180.0
N1^i^—Sn1—N2—C7	180.000 (2)	C4—C5—C6—N1	0.0

Symmetry codes: (i) −*x*+1, −*y*+1, −*z*+1; (ii) −*x*, −*y*+1, −*z*.
